# The Vasomotor Response to Dopamine Is Altered in the Rat Model of l‐dopa‐Induced Dyskinesia

**DOI:** 10.1002/mds.28357

**Published:** 2020-11-02

**Authors:** Samuel Booth, Abdullah Ramadan, Dali Zhang, Lingling Lu, Gilbert Kirouac, Michael F. Jackson, Chris Anderson, Ji Hyun Ko

**Affiliations:** ^1^ Department of Human Anatomy and Cell Science University of Manitoba Winnipeg Manitoba Canada; ^2^ Kleyson Institute for Advanced Medicine Health Science Centre Winnipeg Manitoba Canada; ^3^ Department of Pharmacology and Therapeutics University of Manitoba Winnipeg Manitoba Canada; ^4^ Department of Oral Biology University of Manitoba Winnipeg Manitoba Canada

**Keywords:** Parkinson's disease, blood flow, dyskinesia, dopamine

## Abstract

**Background:**

Levodopa (l‐dopa) is the frontline treatment for motor symptoms of Parkinson's disease. However, prolonged use of l‐dopa results in a motor complication known as levodopa‐induced dyskinesia (LID) in ~50% of patients over 5 years.

**Objectives:**

We investigated neurovascular abnormalities in a rat model of LID by examining changes in angiogenesis and dopamine‐dependent vessel diameter changes.

**Methods:**

Differences in striatal and nigral angiogenesis in a parkinsonian rat model (6‐OHDA lesion) treated with 2 doses of l‐dopa (saline, 2, and 10 mg/kg/day subcutaneous l‐dopa treatment for 22 days) by 5‐bromo‐2'‐deoxyuridine (BrdU)‐RECA1 co‐immunofluorescence. Difference in the vasomotor response to dopamine was examined with 2‐photon laser scanning microscopy and Dodt gradient imaging.

**Results:**

We found that the 10 mg/kg l‐dopa dosing regimen induced LID in all animals (n = 5) and induced significant angiogenesis in the striatum and substantia nigra. In contrast, the 2 mg/kg treatment induced LID in 6 out of 12 rats and led to linearly increasing LID severity over the 22‐day treatment period, making this a promising model for studying LID progression longitudinally. However, no significantly different level of angiogenesis was observed between LID versus non‐LID animals. Dopamine‐induced vasodilatory responses were exaggerated only in rats that show LID‐like signs compared to the rest of groups. Additionally, in juvenile rats, we showed that DA‐induced vasodilation is preceded by increased Ca^2+^ release in the adjacent astrocytes.

**Conclusion:**

This finding supports that astrocytic dopamine signaling controls striatal blood flow bidirectionally, and the balance is altered in LID. © 2020 The Authors. *Movement Disorders* published by Wiley Periodicals LLC on behalf of International Parkinson and Movement Disorder Society.

## Introduction

Levodopa (l‐dopa) is the gold standard treatment for Parkinson's disease (PD). However, with years of l‐dopa treatment, patients variably develop motor complications associated with peak l‐dopa dose termed l‐dopa‐induced dyskinesia (LID). LID patients are distinguished from stable l‐dopa responders by large increases in extracellular dopamine at peak‐dose of l‐dopa, which is normalized off medication.^1^ This increase in dopamine release at peak plasma concentration is also observed in animal models of LID,^2^, ^3^ and animal studies indicated that presynaptic mechanisms of dopamine release are disrupted in LID.^4^

Neurovascular abnormalities have been associated with lid in both human subjects and rat models, including angiogenesis, blood–brain barrier defects, and acute increases in blood flow at peak l‐dopa dose. In the 6‐OHDA lesioned rat model of LID, chronically l‐dopa‐treated animals showed increased angiogenesis in the putamen, globus pallidus, and substantia nigra (SN), which was accompanied by evidence of blood–brain barrier dysfunction.^5^, ^6^, ^7^, ^8^ Angiogenesis may contribute to LID by allowing dysregulated transmission of l‐dopa across the blood–brain barrier in the striatum, exacerbating the large transient flux of synaptic dopamine that is characteristic of LID.^6^, ^9^, ^10^ However, blood–brain barrier deficiencies are independently a hallmark of neurodegenerative diseases including PD. It is therefore unknown whether angiogenesis is necessary for LID and to what extent angiogenesis distinguishes LID subjects from stable l‐dopa responders. An acute increase in blood flow in the striatum has been observed in LID subjects at peak l‐dopa dose, which is normalized when l‐dopa is withdrawn and is independent of glucose metabolism.^11^, ^12^ In normal conditions, regional cerebral blood flow is regulated by local synaptic activity through communication between astrocytes, neurons, and vascular smooth muscle in a process known as neurovascular coupling.^13^, ^14^ These data indicate the neurovascular coupling response is dysregulated in LID. Astrocytes play a key role in the neurovascular coupling response, however, the role of astrocytic changes in cerebral blood flow regulation in LID has not been characterized.

Previously, stimulation of dopaminergic receptors on astrocytes has been shown to induce vessel diameter changes, indicating dopaminergic signaling is an important aspect of the neurovascular coupling response.^15^, ^16^ Dopaminergic signaling can induce astrocyte‐mediated vasodilation^15^, ^17^ and vasoconstriction,^15^, ^18^, ^19^ indicating bimodal mode of action. Given that there is astrocytic dysfunction in LID, and astrocytes are an important part of the neurovascular coupling response, we hypothesized that the neurovascular uncoupling observed may be induced by a change in the astrocytic response to dopaminergic stimulation.

We used a rat model of LID to investigate inter‐subject differences in striatal angiogenesis. For this purpose, we used both a high dose of l‐dopa that induced severe dyskinesia in all animals, as well as a low l‐dopa dose that induced dyskinesia only in a portion of the treated animals. We also used ex vivo imaging to demonstrate that an increase in vasodilation differentiates LID animals from non‐LID animals. Additionally, in juvenile animals, we found that astrocytic calcium signaling dictates the subsequent vasomotor response to dopamine in adjacent astrocytes.

## Materials and Methods

### Animal Model

Based on the previous literature on LID rat model studies, we investigated 84 female Sprague–Dawley rats, housed in a 12‐hour light/dark cycle in pairs with access to food and water ad libitum. All experiments were approved by the University of Manitoba committee on animal care in accordance with Canadian Council on Animal Care guidelines. Sixty‐four animals underwent intracranial injection with 6‐OHDA to produce a unilateral dopamine denervating lesion (for details, see Supplementary Methods).

### Experimental Design

Supplementary Table S1 shows the number of animals in each group for each experiment. To examine the angiogenesis and confirm dopaminergic lesion in LID animal model, 22 animals (weighing 140–160 grams) were used for immunohistochemical analysis using Brdu‐RECA1 co‐immunofluorescence in confocal microscopy. Twelve animals received 2 mg/kg l‐dopa, 5 animals received 10 mg/kg l‐dopa, and 5 animals received an equivalent volume of isotonic saline for 22 days. 5‐Bromo‐2'‐deoxyuridine (BrdU) injection took place in 2 5‐day sessions at days 4–8 and days 14–18 of treatment. At the end of experiments, animals were sacrificed via perfusion and collecting brain tissue. Details on the number of animals per group, daily treatment, immunohistochemistry can be found in the Supplementary Materials.

To examine the dopamine (DA)‐induced vasomotor response in LID animal model, 42 rats (weighing 140–160 grams) were investigated using 2‐photon laser scanning microscopy (TPLSM). Thirty‐two 6‐OHDA lesioned animals were administered 2 mg/kg l‐dopa for 22 days. Five 6‐OHDA lesioned animals were treated with an equivalent volume of isotonic saline as a drug naïve control group, and 5 non‐6‐OHDA lesioned animals were used as a wild‐type control. On day +23, 1 day after the final l‐dopa injection in LID and non‐LID or saline, the animals were decapitated for brain slice preparation and Dodt gradient contrast imaging. The decapitation was obtained without anesthesia because anesthetic agents such as isoflurane affect DA activity,^20^ astrocytic Ca^2+^ activity,^21^ and vasomotor response.^22^, ^23^

To examine the relationship between astrocytic Ca^2+^ and vasomotor responses to DA, an additional 20 rats (21–28 postnatal days) were investigated using TPLSM. Younger rats were used for the examination of extracellular calcium dynamics, because older animal brains did not survive dye incubation. These rats were decapitated in the same manner as described above. All analysis was performed by raters blind to the sample condition.

### Behavioral Tests

After recovering from lesioning, animals were screened for hemi‐parkinsonian‐like behaviors using cylinder test as described elsewhere.^24^ On days 1, 11, and 22 of l‐dopa treatment, an abnormal involuntary movements (AIMs) test was performed to assess LID‐like behavior in the animals. AIMs test was performed by monitoring animals at 20‐minute intervals over a 3‐hour period after a daily intraperitoneal l‐dopa injection. Animals were scored based on axial, orolingual, and limb AIMs on a severity scale of 0–4 as described elsewhere.^25^ AIMs scores in each of the 3 categories for each animal were summed for each session as the total AIMs score. Animals expressing AIMs scores ≥2 of 3 categories by day 22 were classified as LID animals.

### Confocal Microscopy

Confocal laser scanning microscopy was performed using a Zeiss LSM 880 microscope with the accompanying ZEN software package (for detailed setup, see Supplementary Methods). Endothelial cell proliferation is the first stage of vascular sprouting, and histologic confirmation of an increase in BrdU‐labeled endothelial cells is an indicator of angiogenesis.^26^ From each image, angiogenesis was assessed by counting the number of proliferating endothelial cell‐labeled vessels per mm^2^ of tissue. Proliferating endothelial cells were identified as BrdU‐labeled nuclei with a characteristic flattened shape occurring within the RECA1‐labeled vessel. The difference in the number of colocalizations between the lesioned and non‐lesioned hemispheres was used to determine differences in angiogenesis on the lesioned hemisphere.

Imaging of the TH immunolabeled tissue was performed using Zeiss Apotome 2 microscope using the 20× objective. Tile‐scan was used to capture the entire slice. From these images, dopaminergic lesion status was assessed by the number of immunoreactive cell bodies in the SN of the lesioned hemisphere compared to the unlesioned one. Animals with <90% dopaminergic cell loss in the lesioned hemisphere were excluded from the study due to incomplete dopaminergic lesion.

### TPLSM and Dodt Gradient Imaging

The protocol for preparing tissue for imaging of striatal brain slices can be found in the Supplementary Methods. TPLSM imaging scans were performed using a Ti‐sapphire laser with an excitation wavelength of 800 nm and 700 nm/100 ms laser pulse. The edge of the lumen could be identified beneath the thick smooth muscle layer of the arterioles, which was clearly observed in the Dodt scans. After the first 2 minutes of artificial cerebrospinal fluid (aCSF) perfusion (baseline), DA (dopamine hydrochloride; sigma H8502; 500 nM) diluted in aCSF was perfused (2 mL/min). During DA perfusion, the total volume in the recording chamber was consistently set to 1 mL. If there was a measurable widening (≥5%) in the distance between the 2 opposing parallel sides of the vascular lumen, the response was considered as vasodilation. If narrowing in the distance is observed, it was considered as vasoconstriction. If the percentage changes in the distance between the 2 sides is negligible (<5% from the baseline) for 10 minutes of recording time, it was considered as an unchanged response.

Twenty rats at postnatal day (21–28; “juvenile rats”) were used to examine astrocytic calcium dynamics and vessel diameter changes using TPLSM (for details, see Supplementary Methods). To examine the potential differences in different DA dose effect, we have tested 2 different DA concentrations at 500 nM (18 slices) and 20 μM (15 slices). Isolectin B_4_ labels the outer layer of the basement membrane, and thus the vessel diameter was measured on the outer boundaries of the arterioles. The emitted signal was obtained by photomultiplier tubes at 488 nm (isolectin B_4_) and 580 nm (rhod‐2). Recorded images were analyzed every 3 seconds using Prairie View software (Bruker) to determine vessel lumen diameter. Changes in astrocytic calcium were determined by measuring rhod‐2 Ca^2+^ fluorescence intensity changes, which was quantified by measuring the mean value of a manually selected somatic area of astrocytes using ImageJ software.

### Statistical Analysis

All statistical analysis was performed in IBM SPSS 25 software (IBM Corp., Armonk, NY). Differences in angiogenesis between groups were assessed using 2 × 4 repeat measures ANOVA for hemisphere (contralateral and ipsilateral to lesioned side) and group (saline control, 2 mg/kg/day l‐dopa with no LID, 2 mg/kg/day l‐dopa with LID, and 10 mg/kg/day l‐dopa). Between groups, comparisons were performed using Tukey's honest significant difference post‐hoc test. A χ^2^ test was used to examine the frequency of vasodilation, vasoconstriction, and unchanged vessel responses after DA perfusion across different groups (LID, non‐LID, PD control, and wild‐type [WT] control). One‐way ANOVA was used to test if the summed vasomotor changes (regardless of direction) induced by DA were different across groups, followed by post‐hoc Tukey test. In the juvenile rat experiments, unpaired t‐test was used to investigate the effect of 2 different doses of DA on the maximum vasomotor responses. Results were considered statistically significant at *P* < 0.05.

## Results

### Dopaminergic Lesion and Behavioral Tests of LID Rat Model

**FIG. 1:**
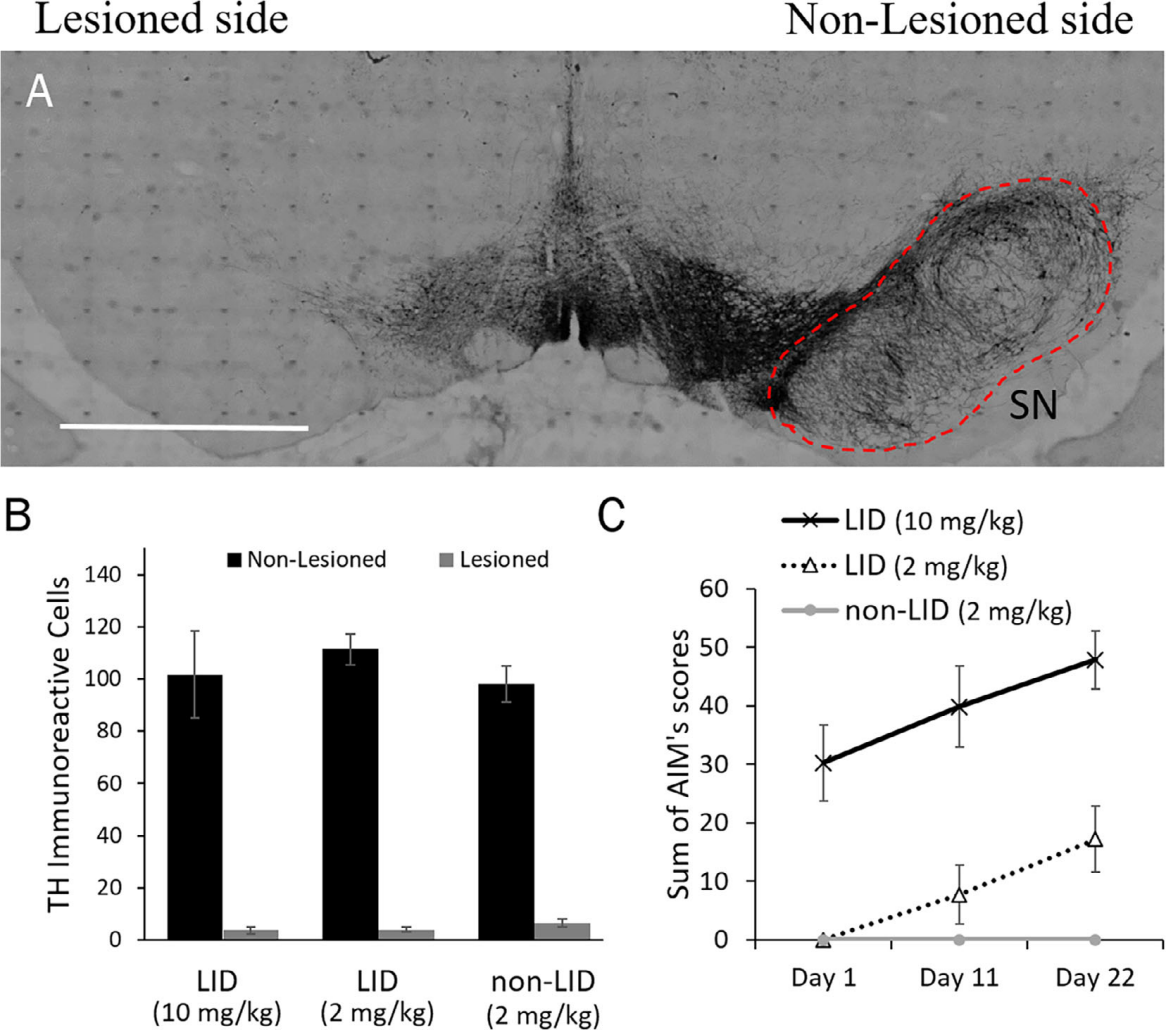
Rat model of parkinsonism/LID using high (10 mg/kg/day) and low (2 mg/kg/day) dosage of l‐dopa. (**A**) An example image of an animal with dopamine denervating lesion, where tyrosine hydroxylase immunoreactivity in the SN is intact on the non‐lesioned hemisphere (right) compared with almost total denervation in the lesioned hemisphere (left). Image produced from an 8 × 20 tiled scan of the entire region using the 20× objective. l, lesioned; nl, non‐lesioned hemisphere. The scale bar indicates 2 mm. (**B**) TH immunoreactivity between each hemisphere in each group, with results expressed as mean ± SD. The lesioned hemisphere shows a large reduction in TH immunoreactivity in the lesioned hemisphere substantia nigra (gray) compared to the unlesioned hemisphere (black). Although there was significant difference in TH immunoreactivity between hemispheres (F[1,34] = 625.34, *P* < 0.001), there was no significant difference in dopaminergic denervation measured by TH immunoreactivity between treatment groups (F[2,34] = 0.807, *P* = 0.456). (**C**) Comparison between sum of AIMs scores on each day of l‐dopa treatment between each treatment group. Both the 10 mg/kg and the 2 mg/kg LID groups progress over time, however, the 10 mg/kg group had strong AIMs scores beginning from day 1 (*P* < 0.001), whereas the 2 mg/kg LID group did not have any AIMs symptoms beginning on treatment day 1. [Color figure can be viewed at wileyonlinelibrary.com]

We confirmed that all 6‐OHDA‐lesioned animals passed cylinder test (>60% forelimb asymmetry; 68.92 ± 14.42, data not shown). Abnormal involuntary movements test were used to quantify LID‐like behaviors. In the 10 mg/kg l‐dopa group, 5 of 5 animals developed LID. Out of the 45 animals in the 2 mg/kg treatment group, 17 animals developed dyskinesia symptoms, whereas 28 animals failed to develop dyskinesia within the 22 days. Two of 64 animals that passed cylinder test (>60% forelimb asymmetry) showed incomplete lesion and thus were excluded from the statistical analysis. There was no significant difference between lesion size between groups subsequently administered 2 mg/kg (LID and non‐LID) or 10 mg/kg l‐dopa F[2,34] = 0.807, *P* = 0.456 (Fig. 1B). Figure 1C shows the sum of axial, orolingual, and limb AIMs from the LID animals on days 1, 11, and 22 of l‐dopa treatment. In the 10 mg/kg l‐dopa group, all animals developed moderate AIMs beginning on the first day of l‐dopa treatment 31.6 ± 9.017. None of the animals in the 2 mg/kg treatment group had AIMs on day 1, however, by day 11, LID‐like behaviors had begun in the 6 LID animals (7.67 ± 6.4704) and peaked at the end of the 22‐day treatment period (17.17 ± 4.07). The other 6 animals (non‐LID; 2 mg/kg) showed no LID‐like behaviors.

### Angiogenesis Is Affected by High Dose but Not Low Dose L‐dopa

**FIG. 2:**
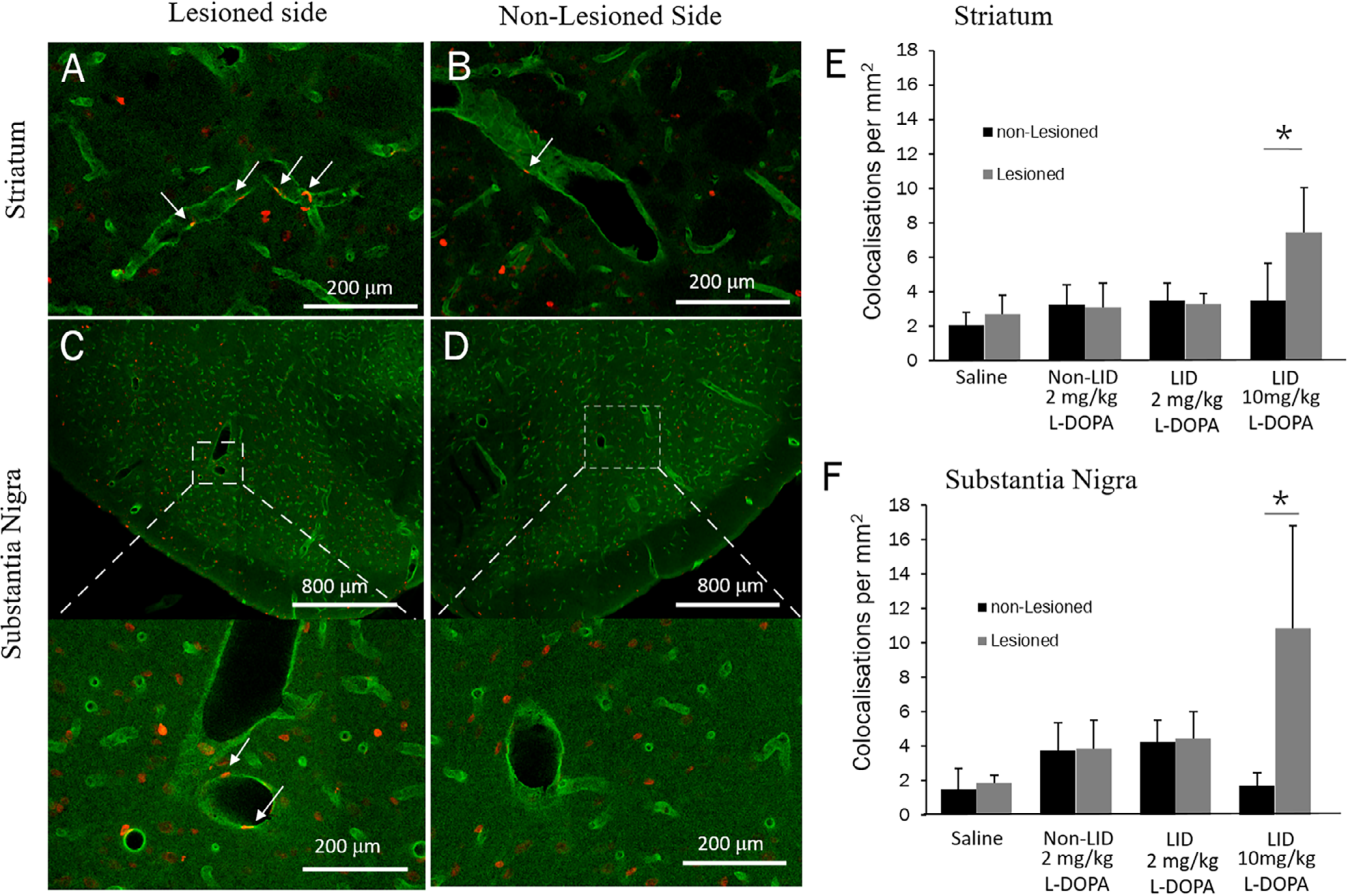
Chronic l‐dopa treatment induces angiogenesis in high dose group but fails to differentiate LID animals from stable l‐dopa responders when a low dose is used. (**A–D**) Laser scanning confocal microscopy of the putamen and the substantia nigra of an animal with dopamine denervating lesion, treated with 10 mg/kg l‐dopa for 22 days. RECA‐1 immunoreactive vessels are green and BrdU immunoreactive nuclei are red. White arrows indicate proliferating endothelial nuclei. Number of BrdU‐RECA‐1 colocalizing nuclei per mm^2^ in the striatum (**E**) and substantia nigra (**F**) were compared between the saline control, 2 mg/kg/day LID, 2 mg/kg/day non‐LID, and 10 mg/kg/day groups in the lesioned and non‐lesioned hemispheres. Statistics comparing lesioned and non‐lesioned hemisphere within each subject using Tukey's honest significant difference post‐hoc test. *P < 0.001. Error bars indicate SD. [Color figure can be viewed at wileyonlinelibrary.com]

To histologically evaluate angiogenesis in the striatum and the SN, we co‐labeled BrdU‐positive cells (indicating proliferating cells) with RECA1, a marker of endothelial cells. We counted the number of BrdU‐labeled nuclei (indicating proliferating cells) within the endothelial wall. In both the striatum and the SN, there was significant interaction effect between group and hemisphere (F[3,18] = 8.193, *P* = 0.001 and F[3,18] = 9.586, *P* = 0.001, respectively) (Fig. 2). We found in the 10 mg/kg group, there was about a 2‐fold increase in the number of BrdU‐RECA1 colocalizations in the striatum and the SN of the lesioned hemisphere compared to the unlesioned hemisphere (*P* < 0.001). However, there was no significant evidence of angiogenesis in the LID animals with 2 mg/kg in either the striatum or substantia nigra (*P* = 0.737 and *P* = 0.927, respectively). This was consistent in the non‐LID animals (*P* = 0.849 and *P* = 0.895 for striatum and SN, respectively). Between groups comparison revealed that when 2 mg/kg l‐dopa dose is used, LID and non‐LID animals could not be differentiated on the basis of angiogenesis in either the striatum or SN (*P* = 0.992 and *P* = 0.945, respectively).

### The Dopamine‐Induced Vasomotor Response in Rat Model of L‐dopa‐Induced Dyskinesia

**FIG. 3:**
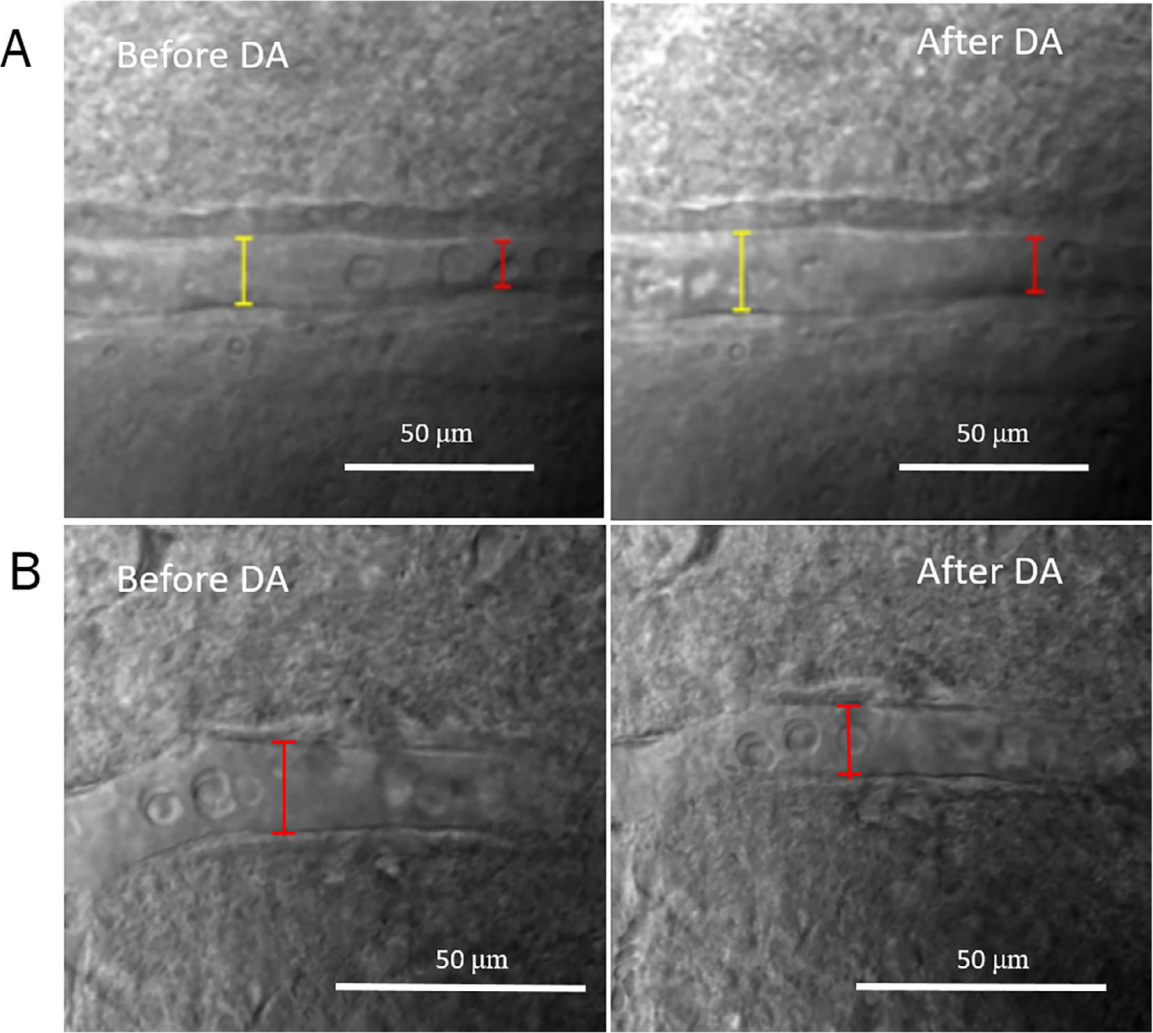
Arteriole vasodilation and vasoconstriction in response to 500 nM dopamine perfusion in striatal brain slices observed by Dodt gradient contrast imaging. (**A**) Arteriole vasodilation trigged by DA from a LID animal. The luminal diameter measurements have been conducted in 2 sites because the vessel was long. The baseline arteriole diameter at the yellow and red bars was 16.5 and 11 μm, respectively. After 10 minutes of DA perfusion, the arteriole diameter increased to 19 μm (yellow bar) and 13 μm (red bar). Scale bar = 50 μm. (**B**) DA induced vasoconstriction in the striatal arteriole from a LID animal. The arteriole diameter during the baseline state was 14.5 μm. The arteriole after DA perfusion was 12.33 μm. Scale bar = 20 μm. The original analysis has been done by Prairie View software. These pictures have been reanalyzed with Image‐J software for visualization purpose. [Color figure can be viewed at wileyonlinelibrary.com]

**FIG. 4:**
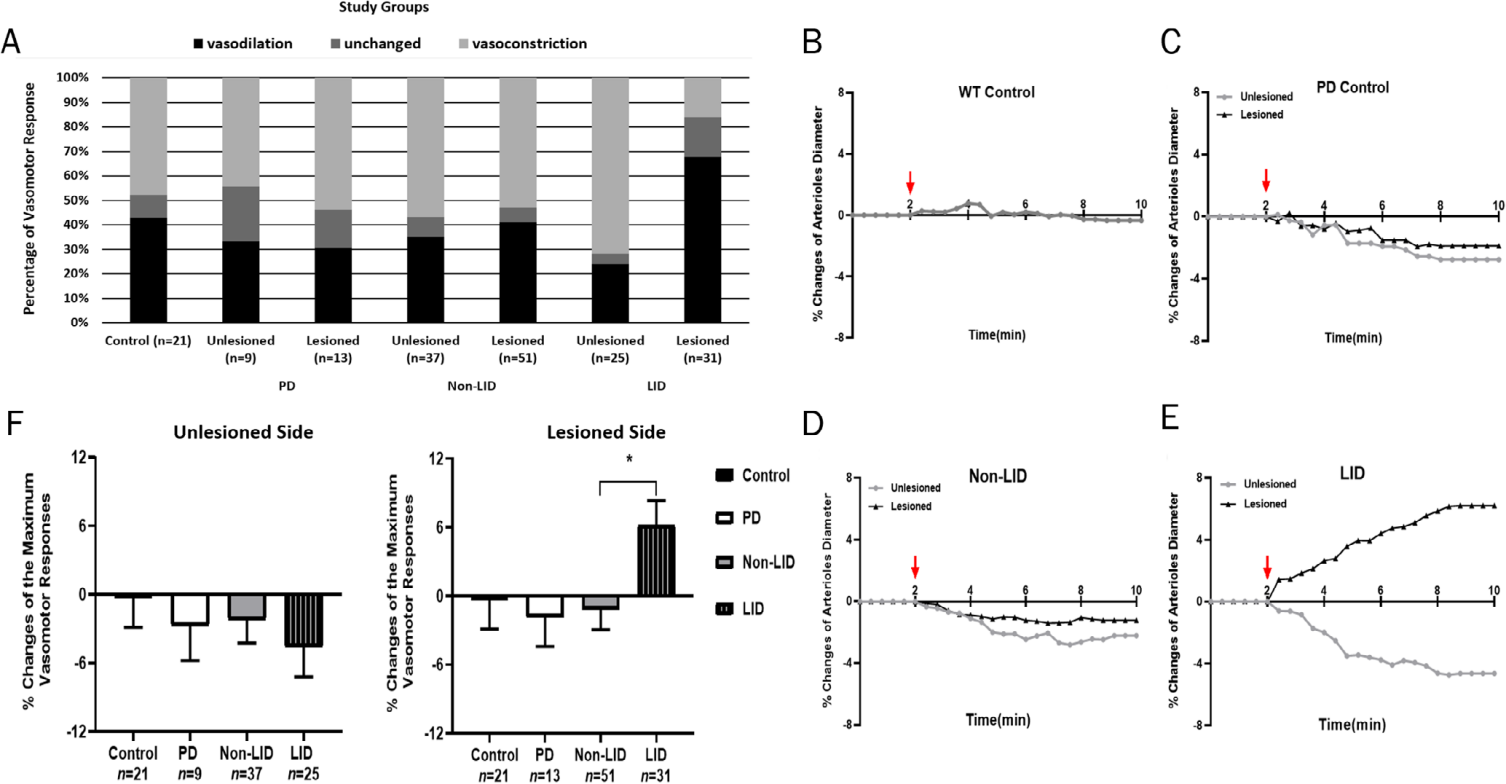
Vasomotor response to DA perfusion in LID animals is significantly shifted toward vasodilation versus vasoconstriction compared to non‐LID animals, and controls. (**A**) Proportion of vessels that showed vasodilation, vasoconstriction, and no change after perfusion (500 nM) of the 4 studied groups. The statistical result of the chi square test is summarized in Supplementary Table S2. (**B**) WT control (5 animals). (**C**) PD control (6‐OHDA lesion, DA naïve; 5 animals). (**D**) Non‐LID (21 animals). (**E**) LID (11 animals). The DA perfusion started after the baseline (the first 2 minutes aCSF perfusion) where the red arrows indicate. (**F**) The summed vasomotor responses in the striatum of the 4 groups for the lesioned and non‐lesioned side. No significant difference was observed in the unlesioned hemispheres (F[3,88] = 0.493, *P* = 0.688, one‐way ANOVA). The maximum vasomotor responses in the lesioned sides were significantly different across the groups (F[3,112] = 2.968, *P* = 0.035). Post‐hoc Tukey test revealed significantly larger vasodilation in LID versus non‐LID (*P* = 0.032). The error bar indicates SD. [Color figure can be viewed at wileyonlinelibrary.com]

To evaluate the effect of dopamine on vasomotor response, we used ex vivo microscopy to image both vasodilation and vasoconstriction evoked by DA perfusion (500 nM) in the arterioles of rat striatal brain slices. Figure 3 shows examples of vasoconstriction and vasodilation in response to DA application were observed using Dodt gradient contrast imaging (Supplementary Videos S1 and S2, respectively). The DA‐induced vasomotor responses of all groups are represented in Figure 4A, whereas the statistical comparison between different conditions are summarized in Supplementary Data Table S2. The percentage of observed arterioles showing vasodilation (43%) and vasoconstriction (48%) response to DA was almost equal in the brain slices of WT control rats. This trend was not significantly altered by 6‐OHDA lesion (PD control) in either side (lesioned and unlesioned, *P* > 0.6, χ^2^). No hemispheric difference (*P* = 0.8, χ^2^) was observed in lesioned animals without l‐dopa treatment. In the lesioned plus 2 mg/kg l‐dopa animals, significantly altered DA‐induced vasomotor response was not observed in non‐LID rats in either hemisphere. However, the DA‐induced vasomotor response in the LID rats was significantly shifted toward vasodilation (68% of observed arterioles) in the lesioned side compared to the unlesioned side (*P* < 0.001, χ^2^), which was significantly different from WT control rats (*P* = 0.048, χ^2^). The time course of vessel diameter changes is visualized in Figure 4B–4E for the different groups. No distinct hemispheric differences were observed in PD control rats nor non‐LID (Fig. 4C,D). However, in the LID animals, DA application induced an increase in vasodilation in the lesioned hemisphere compared to the unlesioned side in the LID rats (Fig. 4E). There was no significant difference in the summed vasomotor changes in the unlesioned side across all groups (F[3,88] = 0.4926, *P* = 0.6883, one‐way ANOVA). However, in the lesioned side, there was a significant group effect (F[3,112] = 2.968, *P* = 0.035, one‐way ANOVA) with a significantly larger vasodilation in the LID versus non‐LID animals (*P* = 0.032, post‐hoc Tukey test) (Fig. 4F). Therefore, dopamine‐induced vasodilatory responses were exaggerated only in rats that showed LID‐like signs compared to non‐LID animals and controls.

### Dopamine Induced Astrocytic Control Over Striatal Vessel Diameter

**FIG. 5:**
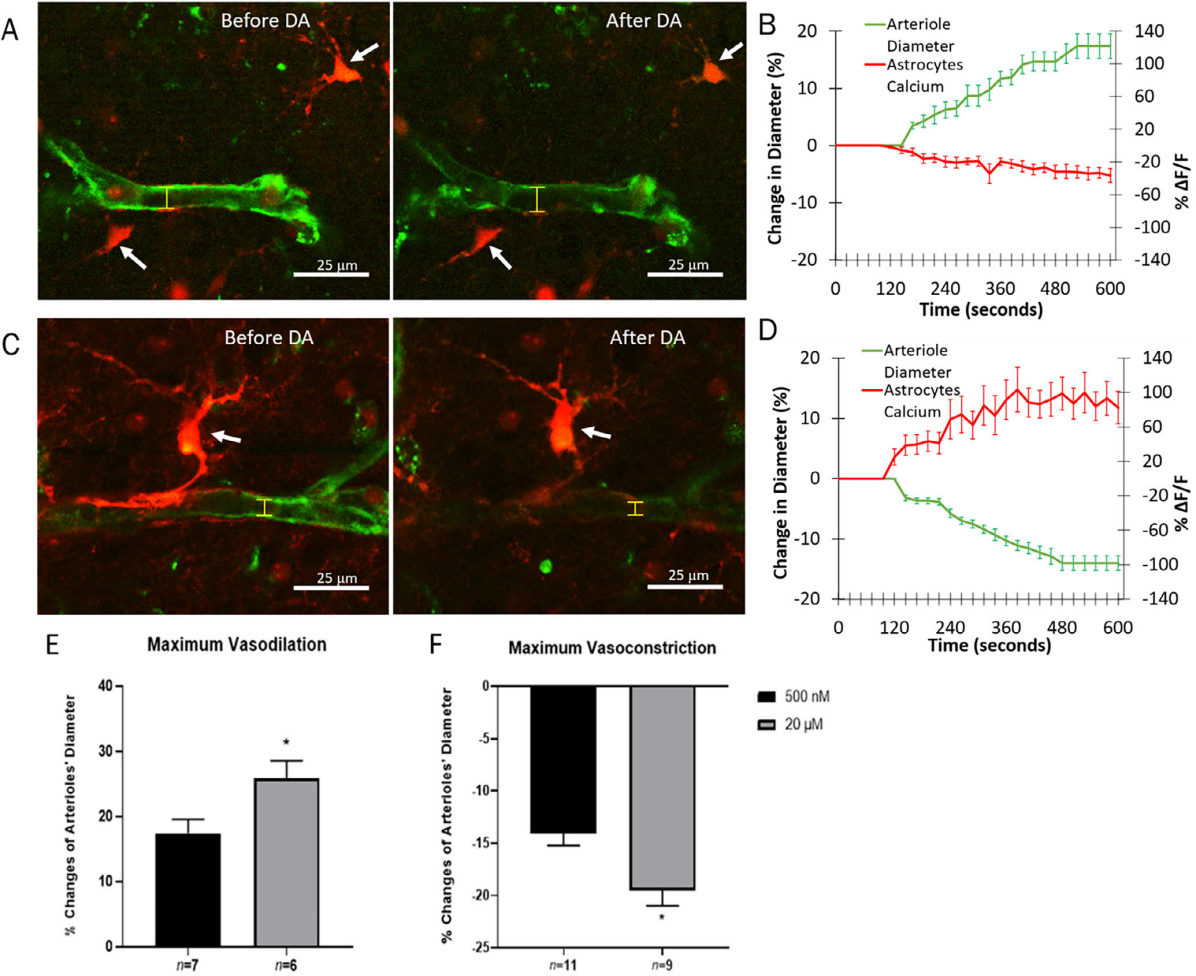
The vasodilation induced by DA is accompanied with calcium reductions in perivascular astrocytes, whereas vasoconstrictive response to DA is corresponded with calcium increases in juvenile rat (21–28 postnatal days) striatal slices. Representative TPLSM images of calcium (red, Rhod 2) in perivascular astrocytes (white arrow) and triggered arteriolar (green, isolectin B4) vasodilation (yellow arrow) (**A**) and vasoconstriction (**C**) are shown before and after DA. The percentage changes in astrocytes Ca^2+^ signaling (green) and arteriole diameter (red) before and after DA during vasodilation (**B**) and vasoconstriction (**D**). Dose‐dependent effect of DA (500 nM and 20 μM concentrations) on the maximum observed increase (**E**) and decrease (**F**) in vessel diameter. Data are presented as mean ± SD. [Color figure can be viewed at wileyonlinelibrary.com]

We used 2‐photon laser scanning microscopy to examine changes in arteriole diameter and astrocytic calcium signaling in brain slices of juvenile rats. Applying DA resulted in a balance between vasodilation and vasoconstriction responses in striatal arterioles in the juvenile animal brain slices. An example of decreased astrocytic Ca^2+^ level (Rhod‐2, AM) and widened striatal arteriole (isolectin B_4_) is shown after DA perfusion (Fig. 5A; see Supplementary Video S3). Interestingly, we found that the vasodilation was preceded by a decrease in Ca^2+^ in the neighboring astrocytes (Fig. 5B). Among the striatal slices that showed vasodilation, significantly larger vasodilation was observed with 20 μM DA dose compared to the 500 nM group (t [10] = 2.460, *P* = 0.034). Similar phenomena were observed among the striatal slices that showed DA‐induced vasoconstriction. In contrast, we observed that the vasoconstriction was preceded by an increase in Ca^2+^ in the neighboring astrocytes. An example of increased astrocytic Ca^2+^ level (Rhod‐2, AM) and narrowed striatal arteriole (isolectin B_4_) is shown after DA perfusion (Fig. 5C,D; see Supplementary Video S4). Examining the time course of vasomotor changes and astrocyte Ca^2+^ signaling, it appears that vasoconstriction was always preceded by an increase in the Ca^2+^ in the adjacent astrocytes (Fig. 5D). Applying different DA concentrations did not result in a different balance in the vasomotor response between vasoconstriction and vasodilation (39% vasodilation in 500 nM DA; 61% vasoconstriction in 500 nM DA; 40% vasodilation in 20 μM DA; 60% vasoconstriction in 20 μM DA). The degree of maximum vasodilation and vasoconstriction was significantly greater with a high DA dose (20 μM) groups compared to the low DA dose (500 nM) group (t [10] = 2.460, *P* = 0.034; t [16] = 2.841, *P* = 0.0116, Fig. 5E,F). We directly observed the dopamine‐induced astrocytic Ca^2+^ activity in relation to these vasomotor changes in the striatum and showed that increases and decreases in astrocytic calcium dictate the subsequent vasoconstriction and vasodilation, respectively.

## Discussion

We replicated the previous findings showing that chronic l‐dopa treatment with 10 mg/kg‐induced angiogenesis in the lesioned hemisphere of 6‐OHDA rat model.^5^ However, when using the lower 2 mg/kg l‐dopa dose, we found no difference in angiogenesis between the LID group and the stable l‐dopa responding animals. This indicates that angiogenesis is not necessary for the induction of mild LID‐like behaviors, but could nevertheless contribute or exacerbate LID symptoms in rats with more severe injury. However, an increased vasomotor response to l‐dopa in the direction of arteriole vasodilation did differentiate LID and non‐LID animals. This indicates that a key factor in the neurovascular uncoupling response in LID is an increased sensitivity of the vasodilatory response. Additionally, in juvenile rat brain slices, we found that the direction of striatal vasomotor responses (vasodilation vs. vasoconstriction) is dictated by preceding perivascular astrocytic Ca^2+^ level changes (decreasing vs. increasing, respectively), indicating a potential role of glial dopaminergic system in vascular abnormality associated with LID.^27^, ^28^, ^29^, ^30^, ^31^, ^32^

Previous perfusion imaging studies in humans have shown an increase in striatal blood flow in LID patients in response to l‐dopa treatment much more than non‐LID patients^11^, ^32^ and in rat models with toxin‐induced parkinsonism.^12^ The increased blood flow was normalized when medication was withdrawn, which indicates that there is a change in the vasomotor response regulated by astrocytes and endothelial smooth muscle, rather than a static change in the microvascular architecture.^11^ Our results are in line with this, because we directly observed an increase in arteriole diameter in striatal brain slices of LID animals when DA was applied. The prevalence of vasomotor alterations in LID patients and animal models observed with multiple modalities leads us to speculate on the role of these alterations in dyskinesia symptoms. If dopaminergic signaling in LID subjects causes an increase in striatal CBF, this would result in greater l‐dopa delivery through the blood supply. Importantly, the blood–brain barrier is disrupted in PD, even independently of apparent angiogenesis,^6^, ^33^, ^34^ and may be particularly disrupted in LID subjects. Increased cerebral blood flow could therefore result in greater unregulated l‐dopa transmission into the striatal microenvironment, resulting in increased DA flux responsible for LID symptoms, as well as further reinforcing astrocyte‐mediated CBF increases in a positive feedback loop.

In our ex vivo analysis of vessel diameter changes using Dodt gradient imaging, we found that DA appears to produce both a vasodilatory and vasoconstrictive effect on striatal arterioles, which is consistent with the bimodal vasoactive effect of dopamine described elsewhere,^15^, ^16^ which mimics the opposite effects of D1/D5 (excitatory) versus D2/D3 (inhibitory) on the neuronal activities. In the non‐LID or saline‐treated rats, DA at 500 nM concentration induced an approximately equal number of vasodilatory and vasoconstrictive responses, potentially via the balanced D1‐like versus D2‐like receptor activation. However, in the LID animals, this balance is shifted toward vasodilation resulting in increased CBF.

Changes in the astrocytic reaction to DA may underlie this shift in the vasomotor response in LID animals. This is supported by our observation that in juvenile rat brain slices, dopamine‐induced vasomotor response was always preceded by intracellular calcium signaling in the perivascular astrocytes. One limitation of this study is that we were not able to investigate this in adult parkinsonian animals due to the toxicity of the Rhodamine dye making the adult brain slices unviable. However, our findings in juvenile animals may be a useful hypothesis generating result, as it shows astrocytic calcium‐dependent dopamine signaling pathways are relevant to CBF alterations. Dopamine‐induced calcium increases and decreases in astrocytes are dependent on the type of dopamine receptor involved, with calcium increases requiring both D_1_‐like and D_2_‐like receptors,^35^, ^36^, ^37^ whereas calcium decreases involve D_2_/D_3_ receptors only.^36^ Dopamine‐dependent increases in astroglial calcium levels are dependent on IP3‐related intracellular storage mechanisms^36^ that are induced by dopaminergic pathways directly through interaction between D_1_ receptor and phospholipase C^35^, ^38^ or through D_1_‐dependent modulation of intracellular NADH signaling.^37^ Astroglial calcium decreases could occur through the modulation of voltage‐gated ion channels, which astroglia express functional copies of.^39^, ^40^, ^41^ Interestingly, activation of D_2_/D_3_ receptors in the nucleus accumbens of rats has been shown to modulate the activity of L‐type voltage‐gated ion channels, reducing calcium levels.^36^, ^42^ Therefore, an increase in astrocytic D_2_/D_3_ expression in the striatum could be relevant to cerebral blood flow changes in LID, although further research on this topic is warranted.

Although we only investigated changes in arteriole diameter in the regulation of vertebral blood flow, capillary pericytes are also known to be an important component of neurovascular coupling.^43^ Dopaminergic axons have been shown to innervate both endothelial smooth muscle as well as capillary pericytes, and therefore may directly affect local cortical blood flow.^44^, ^45^

The present study used a 2 mg/kg l‐dopa dose delivered subcutaneously once daily, which is a lower dose than much of the previous literature that typically use 6–10 mg/kg doses.^3^, ^5^, ^24^, ^25^, ^46^ Our rationale for using a lower dose was to produce a split in LID versus non‐LID animals for the purposes of comparing differences between these 2 groups. In contrast with our results, a previous study has indicated that 2 mg/kg l‐dopa induces negligible AIMs symptoms in 6‐OHDA rats.^47^ However, the longer treatment period in our study (22 days vs. 15 days) could be responsible for this discrepancy.

In conclusion, we show that an aberrant vasomotor response mediated by perivascular astrocytes characterizes dyskinesia symptoms in a Parkinsonian rat model. This response could play an important role LID by increasing the unregulated DA transmission in the striatum which underpins dyskinesia symptoms.

## Author Roles

(1) Research Project: A. Conception, B. Organization, C. Execution; (2). Statistical Analysis: A. Design, B. Execution, C. Review and Critique; (3) Manuscript Preparation: A. Writing of the First Draft, B. Review and Critique.

S.B.: 1B, 1C, 2B, 3A, 3B

A.R.: 1B, 1C, 2B, 3A, 3B

D.Z.: 1B, 1C, 2B, 3B

L.L.: 1B, 1C

G.K.: 1A, 3B

M.F.J.: 1A, 3B

C.A.: 1A, 3B

J.K.: 1A, 1B, 2C, 3B

## Financial Disclosures for all authors (for the preceding 12 months)

The authors have no financial disclosures.

## Supporting information


**Appendix S1.** Supporting Information

Table S1.:

Table S2:.

Video S1.:

Video S2.:

Video S3.:

Video S4.:
