# Supplementary material for: The Vasomotor Response to Dopamine Is Altered in the Rat Model of l‐dopa‐Induced Dyskinesia
Source: Mov Disord. 2020 Nov 2;36(4):938–47. doi: 10.1002/mds.28357 (PMC8246949; doi:10.1002/mds.28357)
Supplement: Supplementary file 1 — Appendix S1. Supporting Information [file MDS-36-938-s001.docx]

Supplementary Methods:

*6-OHDA Lesion Surgery:*

30 minutes prior to surgery, animals received 10 mg/kg of the monamine oxidase inhibitor pargyline hydrochloride by intraperitoneal injection to reduce extrasynaptic breakdown of 6-OHDA [25]. 12.5μg of 6-OHDA was dissolved in 0.2% ascorbic acid/saline at a concentration of 5 mg/mL and injected into the right medial forebrain bundle (stereotactic coordinates relative to the bregma: anteroposterior 2.8 mm, lateral -2.0 mm, dorsoventral -8.6 mm) at a volume of 2.5 μL. 6-OHDA solution was infused using a Hamilton microsyringe over a period of 5 minutes. The solution was allowed to absorb into the tissue for an additional 5 minutes before the needle was withdrawn. Animals were allowed to recover for two weeks before screening for hemiparkinsonian-like behaviors.

*Daily L-DOPA and BrdU Treatment*

Animals receiving L-DOPA also received the aromatic L-amino acid decarboxylase inhibitor benserazide (to prevent peripheral metabolism of L-DOPA) at 15 mg/kg of body weight. Both L-DOPA and benserazide were dissolved together in isotonic saline and administered at a volume of 1.0 mL/kg of body weight by subcutaneous injection each morning of the 22 day treatment period.

Animals were also administered 5-Bromo-2’-deoxyuridine (BrdU) which was dissolved in isotonic saline at a concentration of 25 mg/mL and injected intraperitoneally twice daily at a dose of 50 mg/kg of body weight.

*Tissue Preparation and Immunohistochemistry:*

Animals were sacrificed by anesthetizing with isoflurane and exsanguination by perfusing the left ventricle first with isotonic saline, then 4% PFA solution in saline. Brain tissue was collected and left for 24 hours in 4% PFA solution. Tissue was transferred to 20% sucrose in 0.9% saline solution for 24 hours before being transferred to 30% sucrose in 0.9% saline solution for another 24 hours. Brain tissue was sectioned using a crystate (Leica Biosystems) into 40 μm sections and stored floating in PBS at 4 °C for immunohistochemical analysis.

For chromogenic immunodetection of Tyrosine Hydroxylase (to confirm dopaminergic denervation), free floating immunohistochemistry was performed on rat brain sections which were washed in 0.01 M PBS and blocked for 1 hour in PBS-ST (5% Donkey serum, 0.1% BSA, 0.3% Triton X-100 in 0.01 M PBS). Sections were incubated in mouse anti TH (Sigma Aldrich) in PBS-ST at 1:10000 dilution for 48 hours at 4 °C. Sections were then incubated in PBS-ST containing 1:500 dilution of biotinylated donkey anti-mouse IGF (Jackson Immunoresearch) for 2 hours. Signal was then amplified using the Avadin Biotin Complex method using an ABC Kit (Vector Laboratories) for 45 minutes. Detection of the antibody complexes was performed with 3’-3’ –diaminobenzidine as per manufacturer’s recommendations and incubating for 1-2 minutes. Sections were then mounted onto slides with DPX mounting medium (Sigma Aldrich).

For double immunofluorescence of BrdU and RECA1, free-floating immunohistochemistry was performed on striatum and SN. Sections were washed in 0.01 M PBS +1% Trition X-100 and then incubated in 1M hydrochloric acid at 37 °C for 30 minutes for antigen retrieval. The pH was balanced by washing for 3 minutes in 0.1 M borate, pH 8.5. Sections were blocked for with 0.01 M PBS + 0.2% Triton X-100 with 10% donkey serum for 1 hour at room temperature, before incubating with the sheep anti-RECA1 (abcam) and mouse anti-BrdU (abcam) antibodies at 4 °C for 36 hours at 1:100 dilution. For detection, sections were incubated with donkey anti-mouse IgG conjugated with Alexa 561 (abcam) and donkey-anti-sheep IgG conjugated with Alexa 488 (abcam) for 2 hours at room temperature. The sections were mounted onto slides with Fluoromount-G mounting medium (Southern Biotech).

*Confocal Microscopy*

Images were captured with a 20x plan-apochromat objective in two channels using the 561 nm and 488 nm excitation wavelength. 5 non-continuous slices from the striatum and SN of each animal was analysed by imaging a multipanel region of interest from each hemisphere of the slice. In the striatum, this region was a 4 by 5 tiled section in the middle of the striatum measuring about 3 mm^2^. For the SN, an area of the slice encompassing the entire anatomical region was imaged. Each image was comprised of 8-11 z-stacks of which captured the entire depth of the slice.

*Astrocytic Ca2+ imaging using TPLSM*

TPLSM Ca^2+^ imaging study was only performed with juvenile rats because the toxicity of the Rhodamine dye making the adult brain slices unviable. However, our findings in juvenile animals may be a useful hypothesis generating result, as it shows astrocytic calcium-dependent dopamine signaling pathways are relevant to CBF alterations.

Dissecting and slicing of these animals were done as described in the above except that the NMDG slicing solution was replaced with a standard cutting buffer which was based on sucrose. All buffers recipes can be found elsewhere [33]. The harvested brain slices were transferred to a separate chamber that was filled with aCSF for about 30 minutes for heat recovery and uploaded with two fluorescent dyes: Isolectin B4 (5 µg/ml) that was used to label the blood vessels, and Rhod-2, AM (10 µM, Invitrogen) to detect the Ca^2+^ activity in astrocytes under the effect of DA perfusion. The incubation time was one hour. Slices then were placed in clean aCSF at room temperature, then transferred to the TPLSM recording chamber. At baseline, slices were perfused with oxygenated aCSF (O_2_ 20%).
