# Supplementary material for: The Vasomotor Response to Dopamine Is Altered in the Rat Model of l‐dopa‐Induced Dyskinesia
Source: Mov Disord. 2020 Nov 2;36(4):938–47. doi: 10.1002/mds.28357 (PMC8246949; doi:10.1002/mds.28357)
Supplement: Supplementary file 2 — Table S1.: [file MDS-36-938-s002.docx]

| *Confocal imaging experiments* | *LID status* | *#* |
| --- | --- | --- |
| 6-OHDA + saline | - | 5 |
| 6-OHDA + 2mg/kg L-DOPA | Non-LID | 6 |
| 6-OHDA + 2 mg/kg L-DOPA | LID | 6 |
| 6-OHDA + 10 mg/kg L-DOPA | LID | 5 |
| *Dodt Gradient Imaging experiments* | | |
| Wt control | - | 5 |
| 6-OHDA + saline | - | 4 |
| 6-OHDA + 2 mg/kg L-DOPA | Non-LID | 22 |
| 6-OHDA + 2 mg/kg L-DOPA | LID | 11 |
| *TPLSM experiments* | | |
| Juvenile rats | - | 20 |
