# Supplementary material for: The Vasomotor Response to Dopamine Is Altered in the Rat Model of l‐dopa‐Induced Dyskinesia
Source: Mov Disord. 2020 Nov 2;36(4):938–47. doi: 10.1002/mds.28357 (PMC8246949; doi:10.1002/mds.28357)
Supplement: Supplementary file 3 — Table S2:. [file MDS-36-938-s003.docx]

**Supplementary data Table 1**

Group comparisons in proportion of DA-induced vasomotor responses depicted in Figure 4. (*) p ≤ 0.05, (**) P ≤ 0.01, and (***) P ≤ 0.0001.

| **The compared Condition** | **Chi-Square Value** | ***p*-*Value*** |
| --- | --- | --- |
| **WT Control vs PD Rats**  **(Unlesioned Side)** | **0.918** | ***0.632*** |
| **WT Control vs PD Rats**  **(Lesioned Side)** | **0.604** | ***0.740*** |
| **WT Control vs Non-LID Rats**  **(Unlesioned Side)** | **0.451** | ***0.798*** |
| **WT Control vs Non-LID Rats**  **(Lesioned Side)** | **0.376** | ***0.829*** |
| **WT Control vs LID Rats**  **(Unlesioned Side)** | **2.893** | ***0.235*** |
| **WT Control vs LID Rats**  **(Lesioned Side)** | **6.053** | ***0.048 **** |
| **PD Rats**  **(Unlesioned vs Lesioned Sides)** | **0.242** | ***0.886*** |
| **Non-LID Rats**  **(Unlesioned vs Lesioned Sides)** | **0.416** | ***0.812*** |
| **LID Rats**  **(Unlesioned vs Lesioned Sides)** | **17.911** | ***0.0001 ****** |
| **LID vs PD Rats**  **(Unlesioned Sides)** | **3.485** | ***0.175*** |
| **LID vs PD Rats**  **(Lesioned Sides)** | **6.984** | ***0.030 **** |
| **LID vs Non-LID Rats**  **(Unlesioned Sides)** | **1.545** | ***0.462*** |
| **LID vs Non-LID Rats**  **(Lesioned Sides)** | **11.427** | ***0.003 ***** |
